# Factors associated with improvement or decline in cognitive function after an ischemic stroke in Korea: the Korean stroke cohort for functioning and rehabilitation (KOSCO) study

**DOI:** 10.1186/s12883-016-0780-3

**Published:** 2017-01-10

**Authors:** Jin A. Yoon, Deog Young Kim, Min Kyun Sohn, Jongmin Lee, Sam-Gyu Lee, Yang-Soo Lee, Eun Young Han, Min Cheol Joo, Gyung-Jae Oh, Junhee Han, Minsu Park, Kyung Pil Park, Kyung-Ha Noh, Won Hyuk Chang, Yong-Il Shin, Yun-Hee Kim

**Affiliations:** 1 Department of Rehabilitation Medicine, Pusan National University School of Medicine, Research Institute of Convergence for Biomedical Science and Technology, 20, Geumo-ro, Mulgeum, Yangsan, 626-770 South Korea; 2 Department and Research Institute of Rehabilitation Medicine, Yonsei University College of Medicine, 50-1 Yonsei-ro, Seodaemun-gu, Seoul 120-752 Republic of Korea; 3 Department of Rehabilitation Medicine, School of Medicine, Chungnam National University, 282 Munhwa-ro, Jung-gu, Daejeon, 301-721 Republic of Korea; 4 Department of Rehabilitation Medicine, Konkuk University School of Medicine, 120-1 Neungdong-ro, Hwayang-dong, Gwangjin-gu, Seoul 143-729 Republic of Korea; 5 Department of Physical and Rehabilitation Medicine, Chonnam National University Medical School, 42 Jebong-ro, Donggu, Gwangju, 501-757 Republic of Korea; 6 Department of Rehabilitation Medicine, Kyung-pook National University College of Medicine, 130 Dongdeok-ro, Jung-gu, Daegu, 700-721 Republic of Korea; 7 Department of Rehabilitation Medicine, Jeju University Hospital, University of Jeju College of Medicine, 15 Aran 13-gil, Jeju, 690-767 Republic of Korea; 8 Department of Rehabilitation Medicine, Wonkwang University School of Medicine, 895 Muwang-ro, Iksan, Jeonlabuk-do, 570-711 Republic of Korea; 9 Research And Statistical Support, Research Institute of Convergence for Biomedical Science and Technology, Pusan National University Yangsan Hospital, 20, Geumo-ro, Mulgeum-eu, Yangsan, 626-770 South Korea; 10 Department of Neurology, Pusan National University College of Medicine, Pusan National University Yangsan Hospital, 20, Geumo-ro, Mulgeum-eu, Yangsan, 626-770 South Korea; 11 Department of Physical and Rehabilitation Medicine, Center for Prevention and Rehabilitation, Heart Vascular and Stroke Institute, Samsung Medical Center, Sungkyunkwan University School of Medicine, 50 Ilwon-dong, Gangnam-gu, Seoul 135-710 Republic of Korea; 12 Department of Rehabilitation Medicine, Pusan National University School of Medicine, Research Institute for Convergence of Biomedical Science and Technology, Pusan National University Yangsan Hospital, 20, Geumo-ro, Mulgeum, Yangsan, 626-770 South Korea

**Keywords:** Ischemic stroke, Cognition, Inverter, Reverter, Risk factors

## Abstract

**Background:**

We conducted a prospective cohort study to investigate prevalence of poststroke cognitive impairment at 3 and 12 months after stroke onset and identify clinical and demographic factors associated with improvement or decline in cognitive function between 3 months and 12 months.

**Methods:**

We analyzed the cognitive assessments of total patients and patients older than 65 years separately. All patients with an ischemic stroke were divided into normal cognitive group (NCG) and impaired cognition group (ICG) by using a cutoff score on the Korean Mini-Mental State Examination (K-MMSE). Patients were additionally classified into 3 subgroups according to the changes in their K-MMSE scores between 3 and 12 months: Stable group with K-MMSE scores changes ranging from −2 to +2 points (−2 ≤ △MMSE ≤ +2); converter group with increase more than 3 points (3 ≤ △MMSE); and reverter group with decrease more than 3 points (−3 ≤ △MMSE). We also analyzed factors affecting cognitive change from 3 months to 12 months among the 3 groups including baseline medical record, stroke and treatment characteristics, and various functional assessments after 3 months.

**Results:**

This study included 2,625 patients with the first time ischemic stroke. Among these patients, 1,735 (66.1%) were classified as NCG, while 890 patients (33.9%) were belonged to the ICG at 3 month. Within the NCG, 1,460 patients (82.4%) were stable group, 93 patients (5.4%) were converter group, and 212 patients (12.2%) were reverter group at 12 months onset. Within the ICG group, 472 patients (53.0%) were stable group, 321 patients (36.1%) were converter group, and 97 patients (10.9%) were reverter group. When different factors were investigated, the three subgroups in NCG and ICG showed significant different factors affecting cognitive function from 3 to 12 month.

**Conclusions:**

The prevalence of cognitive impairment showed difference between 3,12 months. To analyze the cognitive change from 3 month to 12 month, the proportion stable group was dominant in NCG and converter group was higher in ICG. By investigating the influencing factors from each group, we were able to identify the predictors including the age factor.

## Background

Cerebrovascular stroke is considered one of the main causes of dementia [1–3]. It may decrease quality of life in addition to causing other neurological deficits [4]. Post-stroke dementia is defined as a presence of dementia identified at 3 months after an acute stroke [5]. Reasons for a stroke patient to develop dementia are still insufficiently understood. It is not always a direct consequence of cerebrovascular lesions, and, in some cases, post-stroke dementia has a progressive course, suggesting a degenerative rather than a vascular origin [6, 7]. In previous autopsy series, 10 to 15% of dementias occurring after a stroke were found to be due to a combination of vascular and Alzheimer’s disease [8, 9].

Despite consensus that strokes are associated with an increased risk of post-stroke dementia, the data regarding prevalence at 3 months post-stroke are still conflicting, with reports ranging from 6% to more than 50% [10–14]. In addition, cognitive function may vary (either improve or decline) years after a stroke. Snaphaan et al. reported that post-stroke memory dysfunction varied from 23 to 55% at 3 months after a stroke, and this declined from 11 to 31% at 1 year after a stroke. Declined cognitive function may change. A previous cohort study showed 33% of patients with mild cognitive impairments diagnosed in the first 6 months after a stroke showed improvement at 1 year. Several prospective studies have identified delayed improvement in cognitive function after strokes using various diagnostic assessment tools for dementia [15-17].

The pathophysiology of delayed cognitive change after a stroke is multifactorial, and the prevalence rate of post-stroke dementia is higher among older patients [18, 19]. Previous studies have tracked cognitive changes to identify the factors associated with delayed improvement or a decline in cognitive function after stroke. However, no large-scale study has been conducted to investigate the pattern of post-stroke cognitive changes, identify the risk factors, or compare age-related differences using repeated administration of the most commonly used screening tool.

Therefore, we conducted a prospective cohort study in conjunction with the Korean Stroke Cohort for Functioning and Rehabilitation (KOSCO) to identify 1) the prevalence of delayed cognitive impairment; patients progress to either converter, stable, or reverter group after ischemic stroke and 2) clinical and demographic factors associated with improvement or decline in cognitive function between 3 months and 12 months after ischemic stroke. The present study is the first to involve a large and well-characterized Korean cohort, a battery of short cognitive and functional assessments, and a 1-year follow-up.

## Methods

### Study design

KOSCO is a large, multi-centered, prospective cohort study of all acute, first-time stroke patients admitted to participating hospitals in nine distinct areas of Korea. A written informed consent is obtained from all patients prior to inclusion in the study, and the study protocols were approved by the ethics committee of each hospital. The detailed rationale and protocols of KOSCO were described in a previous article [20].

### Study subjects

All consecutive patients with an acute, first-time IS admitted to the representative hospitals were asked to participate in the study. The inclusion criteria were: 1) first-time ischemic stroke with corresponding lesion on a MRI/A scan, 2) age ≥19 years at stroke onset, and 3) onset of symptoms within 7 days prior to inclusion. Exclusion criteria were: 1) recurrent stroke; 2) hemorrhagic stroke; 3) traumatic intracerebral hemorrhage; 4) previously diagnosed dementia or cognitive impairment; 5) persistent aphasia; and 6) history of systemic diseases known to involve the central nervous system.

### Procedure

All eligible patients were recruited from August 2012 to April 2015 at the time of stroke evaluation. After providing a written informed consent, patients were formally enrolled in the study. If a patient was unable to provide informed consent, the consent was obtained from the patient’s legally authorized representative.

### Demographic and clinical characteristics

Baseline demographic and clinical characteristics of enrolled patients were evaluated at 3 months. A complete enumeration survey of all patients was performed using a review of medical records upon the first admission. Survey items included demographic data and presence of cerebrovascular risk factors using standardized, structured questionnaires. The items were classified according to the current guidelines of the American Heart Association [21]. Comorbidities were assessed using the Charlson comorbidity index [22]. Initial stroke severity was recorded at the time of hospital arrival using the Korean National Institute of Health Stroke Scale (K-NIHSS) for ischemic strokes [23]. Physical examination findings and laboratory measures were also recorded. The course of the disease during admission was documented including information on medication use, treatments such as intravenous or arterial thrombolysis, and complications. Patients that received rehabilitation at 3 months were transferred to the rehabilitation center to initiate active rehabilitation after acute management at the neuroscience center. The remaining patients that did not receive any rehabilitation treatments were discharged or transferred to other hospitals instead of being transferred to the Rehabilitation Medicine Department.

### Classification of ischemic stroke; etiology, and neuroimaging

The etiologies of ischemic strokes were classified according to the TOAST criteria [24]. Etiology was determined based on neuro-imaging, medical history, and use of medication. MRI scans were reviewed by neuroimaging specialists in each institute. Ischemic strokes were classified according to arterial territory and as either lacunar or territorial.

### Cognitive assessment

**Fig. 1:**
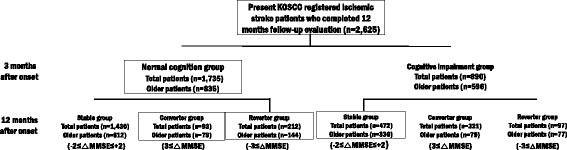
Flow diagram of this study

To identify influencing factors by age, we analyzed the Korean Mini-Mental State Examination (K-MMSE) at 3 months separately between total patients and patients older than 65 years. To analyze changes in cognitive function, all patients were divided into normal cognitive group (NCG) and impaired cognition group (ICG) by using standard deviation score after correcting raw scores by age, sex and education level of the patients [25]. Patients were again classified according to the changes in their K-MMSE scores between 3 and 12 months after stroke onset into stable groups (NCG-SG, ICG-SG) with K-MMSE changes ranging from −2 to +2 points (−2 ≤ △MMSE ≤ +2), converter groups (NCG-CG, ICG-CG) with increases of K-MMSE more than 3 points (3 ≤ △MMSE), and reverter groups (NCG-RG, ICG-RG) with score decreases of K-MMSE more than 3 points (−3 ≤ △MMSE). Factors affecting cognitive change from 3 months to 12 months including baseline medical record, stroke and treatment characteristics, and various functional assessments after 3 months were analyzed among the groups (Fig. 1).

### Functional assessment battery

At 3 months after stroke onset, a face-to-face functional assessment was performed. Assessments included the K-NIHSS for stroke severity, Functional Independence Measure (FIM) [26], Korean modified Barthel Index (K-MBI) [27] for activities of daily living, Fugl-Meyer Assessment (FMA) [28] for motor function, Functional Ambulatory Category (FAC) [29] for mobility and gait, mRS (modified rankin scale) [30] for general functional level, Geriatric depression scale-short form (GDS-SF) [31] for mood, and Euro Quality of Life (EQ)-5D [32] for quality of life (Table 1).

**Table 1:** Functional assessments at 3 months

Domain	Assessment
Stroke severity	National Institute of Health Stroke Scale (NIHSS)
Activities of daily living	Korean modified Barthel Index (K-MBI)
	Functional Independence Measure (FIM)
Cognition function	Korean Mini-Mental State Examination (K-MMSE)
Motor function	Fugl-Meyer Assessment (FMA)
	Modified Ashworth scale (mRS)
Mobility function	Functional Ambulatory Category (FAC)
Mood	Geriatric depression scale-short form (GDS)
Quality of life	Euro Quality of Life-5D

### Statistical analysis

For statistical analysis, we used descriptive statistics for the demographic and clinical characteristics, initial stroke features and treatment methods. Nominal and ordinal data obtained from a baseline review of medical records and initial stroke features were compared using frequency analysis. Scale factors were analyzed using average analysis. Chi-square test and one-way ANOVA were used to compare the influencing factors among groups. Bonferroni correction was done for post-hoc analysis of ANOVA. Statistical analysis was completed using SPSS for Windows version 21.0 (SPSS Inc., Chicago, IL). *P* < 0.05 is considered statistically significant.

## Results

**Fig. 2:**
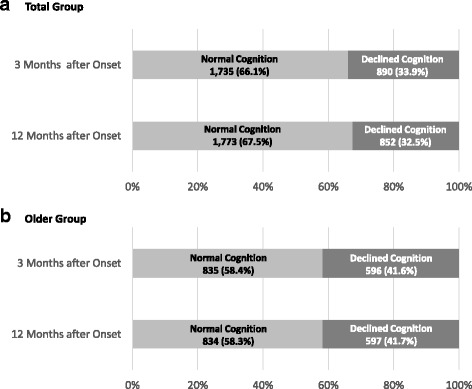
Cognitive function of patients at 3 months and 12 months after stroke onset

**Fig. 3:**
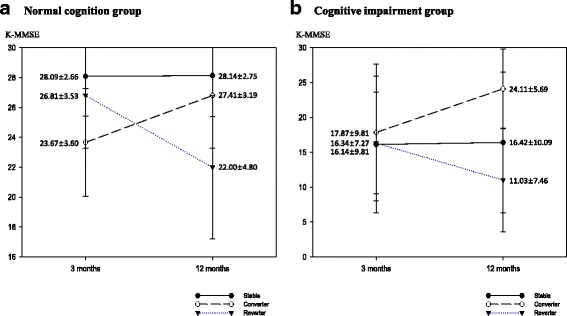
Cognitive change of total patients from 3 months to 12 months

**Fig. 4:**
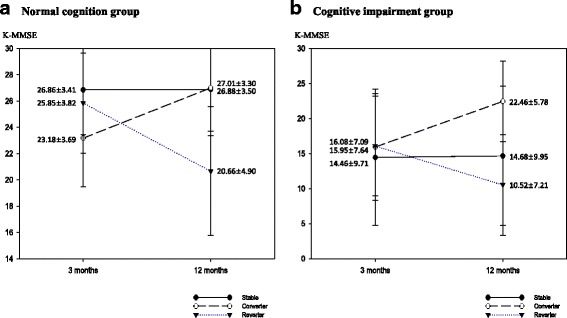
Cognitive change of the older patient group from 3 months to 12 months

A total of 2,625 patients (older patients = 1,431) with first time ischemic stroke were included in this study. Among these patients, 1,735 (66.1%) (older patients = 835 (58.4%)) were classified as NCG, while 890 patients (33.9%) (older patients = 596 (41.6%)) were the ICG at 3 month K-MMSE assessment. Although, percentage of normal and declined cognitive function was similar for older patients at 3 months and 12 months, percentage of normal cognition group was slightly increased and percentage of declined cognition groups was decreased in total patients (Fig. 2). Among NCG, 1,460 (82.4%) (older patients = 612 (73.3%)) were stable group, 93 patients (5.4%) (older patients = 79 (9.5%)) were converter group, and 212 patients (12.2%) (older patients = 144(17.2%)) were reverter group at 12 months onset. Among ICG, 472 patients (53.0%) (older patients = 336 (56.4%)) were stable group, 321 patients (36.1%) (older patients = 183(30.7%)) were converter group, and 97 patients (10.9%) (older patients = 77 (12.9%)) were reverter group (Table 2) (Figs. 3, 4). To analyze the cognitive change from 3 month to 12 month, the proportion stable group was dominant in NCG and converter group was higher in ICG.

**Table 2:** Cognitive change divided by age from 3 months to 12 months

Age	Group	n (%)	Cognitive Change		3 months after onset	12 months after onset
			n (%)		K-MMSE (mean ± SD)	K-MMSE (mean ± SD)
Total Subjects (*n* = 2,625)	Normal Cognition	1,735 (66.1)	Stable	1,430 (82.4)	28.09 ± 2.66	28.14 ± 2.75
			Converter	93 (5.4)	23.67 ± 3.60	27.41 ± 3.19
			Reverter	212 (12.2)	26.81 ± 3.53	22.00 ± 4.80
	Declined Cognition	890 (33.9)	Stable	472 (53.0)	16.14 ± 9.81	16.42 ± 10.09
			Converter	321 (36.1)	17.87 ± 9.81	24.11 ± 5.69
			Reverter	97 (10.9)	16.34 ± 7.27	11.03 ± 7.46
Older age (*n* = 1,431)	Normal Cognition	835 (58.4)	Stable	612 (73.3)	26.86 ± 3.41	26.88 ± 3.50
			Converter	79 (9.5)	23.18 ± 3.69	27.01 ± 3.30
			Reverter	144 (17.2)	25.85 ± 3.82	20.66 ± 4.90
	Declined Cognition	596 (41.6)	Stable	336 (56.4)	14.46 ± 9.71	14.68 ± 9.95
			Converter	183 (30.7)	15.95 ± 7.64	22.46 ± 5.78
			Reverter	77 (12.9)	16.08 ± 7.09	10.52 ± 7.21

*n*, Number; *SD*, Standard Deviation; *K*-*MMSE*, Korean Mini Mental State Examination

Among NCG of total patients, hypertension, and cortical or multiple level involvement was dominant in reverter group, male sex, lower onset age, higher education level were dominant in stable group. In addition, functional assessments in stable group including NIHSS, mRS, FIM, K-MBI, FAC, GDS, and EQ-5D at 3 months were significantly better in scores compared to other groups. For the tendency of ICG of total patients, onset age, hypertension history was higher, education level was lower in reverter group. All functional assessments at 3 months showed better scores in converter groups and worse scores in reverter group (Table 3). Among separated older patients, male sex, lower onset age were dominant in stable group, educational level was lower in reverter group. Functional assessments including NIHSS, mRS, FIM, K-MBI, FAC, GDS, and EQ-5D, at 3 months showed better scores in stable group compared to others. In addition, proportion of receiving rehabilitation therapy at 3 months was lower and all functional l assessments showed better scores in converter group compared to others (Table 4).

**Table 3:** Factors affecting cognitive change from 3 months to 12 months in total patients

Parameters	Normal Cognition (*n* = 1,735)				Declined Cognition (*n* = 890)			
	Stable (*n* = 1,430)	Converter (*n* = 93)	Reverter (*n* = 212)	*P* value	Stable (*n* = 472)	Converter (*n* = 321)	Reverter (*n* = 97)	*P* value
1) Baseline medical record assessments								
Male, n (%)	991 (69.3)	46 (49.5)	107 (50.5)	.000^**^	248 (52.5)	185 (57.6)	52 (53.6)	.362
Age, (mean ± SD)	61.52 ± 12.53_**a**_	69.44 ± 11.16_**b**_	73.75 ± 8.85_**c**_	.000^**^	70.32 ± 11.07_**a**_	66.34 ± 11.54_**b**_	72.32 ± 9.45_**ab**_	.000^**^
Education, n (%)								
Uneducated	114 (8.0)	25 (26.9)	44 (20.8)	.000^**^	35 (7.4)	11 (3.4)	13 (13.4)	.000^**^
0–3 years	46 (3.2)	10 (10.8)	12 (5.7)		40 (8.5)	26 (8.1)	11 (11.3)	
4–6 years	189 (13.2)	25 (26.9)	52 (24.5)		117 (24.8)	73 (22.7)	27 (27.8)	
7–9 years	222 (15.5)	17 (18.3)	45 (21.2)		113 (23.9)	70 (21.8)	21 (21.6)	
10–12 years	468 (32.7)	8 (8.6)	36 (17.0)		135 (28.6)	93 (29.0)	19 (19.6)	
13 years–	391 (27.3)	8 (8.6)	23 (10.8)		32 (6.8)	48 (15.0)	6 (6.2)	
BMI (kg/m^2^), (mean ± SD)	24.10 ± 3.28_**a**_	23.59 ± 3.61_**ab**_	23.49 ± 3.33_**b**_	.023^*^	23.35 ± 3.08	23.47 ± 3.30	22.62 ± 3.27	.071
Risk factors of stroke, n (%)								
Hypertension	779 (54.5)	63 (67.7)	118 (88.7)	.030^*^	275 (58.3)	178 (55.5)	68 (70.1)	.037^*^
Diabetes Mellitus	206 (14.4)	15 (16.1)	24 (11.3)	.412	70 (14.8)	44 (13.7)	22 (22.7)	.091
Coronary heart disease	98 (6.9)	9 (9.7)	15 (7.1)	.587	40 (8.5)	20 (6.2)	5 (5.2)	.339
Atrial fibrillation	118 (8.3)	14 (15.1)	27 (12.7)	.014^*^	68 (14.4)	37 (11.5)	11 (11.3)	.433
Hyperlipidemia	246 (17.2)	12 (12.9)	39 (18.4)	.493	54 (11.4)	42 (13.1)	15 (15.5)	.505
Obesity	190 (13.3)	14 (15.1)	23 (10.8)	.522	49 (10.4)	40 (12.5)	4 (4.1)	.063
Family history	135 (9.4)	5 (5.4)	20 (9.4)	.420	48 (10.2)	26 (8.1)	7 (7.2)	.483
Smoking, n (%)								
Current smokers	462 (32.3)	17 (18.3)	44 (20.8)	.000^**^	127 (26.9)	87 (27.1)	29 (29.9)	.859
Former smokers	202 (14.1)	10 (10.8)	19 (9.0)		55 (11.7)	39 (12.1)	8 (8.2)	
Never smokers	766 (53.6)	66 (71.0)	149 (70.3)		290 (61.4)	195 (60.7)	60 (61.9)	
Alcohol consumption, n (%)								
None	793 (55.5)	62 (66.7)	144 (67.9)	.002^**^	327 (69.3)	202 (63.0)	67 (69.1)	.253
Moderate	452 (31.6)	23 (24.7)	42 (19.8)		97 (20.6)	72 (22.4)	21 (21.6)	
Heavy	185 (12.9)	8 (8.6)	26 (12.3)		48 (10.2)	47 (14.6)	9 (9.3)	
2) Stroke characteristics								
Ischemic type (TOAST)								
Large–artery atherosclerosis	641 (44.8)	58 (62.4)	101 (47.6)	.062	229 (48.5)	173 (53.9)	56 (57.7)	.572
Small-artery occlusion	373 (26.1)	14 (15.1)	53 (25.0)		85 (18.0)	57 (17.8)	15 (15.5)	
Cardioembolism	156 (10.9)	6 (6.5)	24 (11.3)		74 (15.7)	39 (12.1)	9 (9.3)	
Other determined	115 (8.0)	4 (4.3)	12 (5.7)		29 (6.1)	14 (4.4)	5 (5.2)	
Undetermined ischemic stroke	145 (10.1)	11 (11.8)	22 (10.4)		55 (11.7)	38 (11.8)	12 (12.4)	
Ischemic location								
Rt. hemisphere	680 (47.6)	46 (49.5)	99 (46.7)	.580	178 (37.7)	117 (36.4)	41 (42.3)	.698
Lt. hemisphere	648 (45.3)	37 (39.8)	94 (44.3)		262 (55.5)	185 (57.6)	48 (49.5)	
Both hemisphere	102 (7.1)	10 (10.8)	19 (9.0)		178 (37.7)	19 (5.9)	8 (8.2)	
Affected level								
Cortical level	440 (30.8)	37 (39.8)	70 (33.0)	.007^**^	191 (40.5)	139 (43.3)	41 (42.3)	.878
Subcortical level	454 (31.7)	22 (23.7)	49 (23.1)		115 (24.4)	83 (25.9)	25 (25.8)	
Brainstem level	330 (23.1)	13 (14.0)	58 (27.4)		63 (13.3)	41 (12.8)	14 (14.4)	
Multiple level	206 (14.4)	21 (22.6)	35 (16.5)		103 (21.8)	58 (18.1)	17 (17.5)	
3) Treatment characteristics								
IV thrombolysis	104 (7.3)	7 (7.5)	13 (6.1)	.826	43 (9.1)	29 (9.0)	15 (15.5)	.136
IA thrombolysis	21 (1.5)	3 (3.2)	4 (1.9)	.404	23 (4.9)	9 (2.8)	2 (2.1)	.208
IV heparin	121 (8.5)	5 (5.4)	17 (8.0)	.573	33 (7.0)	18 (5.6)	10 (10.3)	.271
Antiplatelet agent	1,140 (79.7)	77 (82.8)	166 (78.3)	.668	333 (70.6)	240 (74.8)	65 (67.0)	.241
Rehabilitation Therapy	386 (27.0)	29 (31.2)	59 (27.8)	.669	187 (60.4)	116 (36.1)	43 (44.3)	.311
Cognitive Therapy	56 (3.9)	4 (4.3)	9 (4.2)	.961	28 (5.9)	33 (10.3)	11 (11.3)	.041^*^
4) Functional assessments								
Cognitive function (K-MMSE)								
3 months from onset	28.09 ± 2.66_**a**_	23.67 ± 3.60_**b**_	26.81 ± 3.53_**c**_	.000^**^	16.14 ± 9.81_**a**_	17.87 ± 7.81_**b**_	16.34 ± 7.27_**ab**_	.024^*^
12 months from onset	28.14 ± 2.75_**a**_	27.41 ± 3.19_**a**_	22.00 ± 4.80_**b**_	.000^**^	16.42 ± 10.09_**a**_	24.11 ± 5.69_**b**_	11.03 ± 7.46_**c**_	.000^**^
Variation (from 3 months to 12 months)	0.05 ± 1.07_**a**_	3.41 ± 1.23_**b**_	−4.81 ± 2.91_**c**_	.000^**^	0.29 ± 1.09_**a**_	6.25 ± 4.19_**b**_	−5.31 ± 2.81_**c**_	.000^**^
Stroke Severity, ADL, Motor, Gait, Depression, QoL (3 months from onset)								
NIHSS	0.77 ± 1.85_**a**_	1.11 ± 2.03_**ab**_	1.12 ± 2.02_**b**_	.015^*^	5.09 ± 6.86_**a**_	3.12 ± 4.62_**b**_	5.25 ± 4.63_**a**_	.000^**^
mRS	0.99 ± 1.02_**a**_	1.44 ± 1.28_**b**_	1.43 ± 1.20_**b**_	.000^**^	2.57 ± 1.68_**a**_	2.17 ± 1.52_**b**_	3.15 ± 1.29_**c**_	.000^**^
FIM	120.00 ± 12.73_**a**_	114.16 ± 17.39_**b**_	113.93 ± 18.05_**b**_	.000^**^	88.70 ± 38.13_**a**_	100.18 ± 30.85_**b**_	82.80 ± 30.61_**a**_	.000^**^
K-MBI	95.44 ± 11.90_**a**_	90.03 ± 16.88_**b**_	89.88 ± 17.34_**b**_	.000^**^	69.11 ± 36.99_**a**_	79.71 ± 28.65_**b**_	63.41 ± 30.94_**a**_	.000^**^
FMA	94.11 ± 15.80	91.19 ± 18.70	89.88 ± 17.34	.119	72.75 ± 35.71_**a**_	81.68 ± 30.77_**b**_	63.65 ± 35.07_**c**_	.000^**^
FAC	4.66 ± 0.87_**a**_	6.59 ± 4.07_**b**_	6.74 ± 4.40_**b**_	.000^**^	3.10 ± 2.03_**a**_	3.65 ± 1.77_**b**_	2.66 ± 1.83_**a**_	.000^**^
GDS	4.95 ± 3.79_**a**_	6.59 ± 4.07_**b**_	6.74 ± 4.40_**b**_	.000^**^	7.25 ± 4.11_**a**_	7.30 ± 4.32_**a**_	9.31 ± 3.92_**b**_	.006^**^
EQ-5D	0.78 ± 0.28_**a**_	0.69 ± 0.33_**b**_	0.65 ± 0.36_**b**_	.000^**^	0.44 ± 0.41_**a**_	0.53 ± 0.40_**b**_	0.31 ± 0.37_**c**_	.000^**^

*n*, Number; *SD*, Standard Deviation; *BMI*, Body Mass Index; *TOAST*, Trial of Org 10172 in Acute Stroke Treatment; *Rt*, Right; *Lt*, Left; *IV*, Intra-Venous; *IA*, Intra-Artrial; *K*-*MMSE*, Korean Mini Mental State Examination; *NIHSS*, National Institutes of Health Stroke Scale; *mRS*, modified Rankin Scale; *ADL*, Activity of Daily Living; *FIM*, Functional Independence Measure; *K-MBI*, Korean version of Modified Barthel Index; *FMA*, Fugl-Meyer Assessment; *FAC*, Functional Ambulation Categories; *GDS*, Geriatric Depression Scale; *QoL*, Quality of Life; *EQ-5D*, EuroQol-5D

^*^ *p* < 0.05; ^**^ *p* < 0.01

_**abc**_ Post HOC group

**Table 4:** Factors affecting cognitive change from 3 months to 12 months in older patients

Parameters	Normal Cognition (n = 835)				Declined Cognition (*n* = 596)			
	Stable (*n* = 612)	Converter (*n* = 79)	Reverter (*n* = 144)	*P* value	Stable (*n* = 336)	Converter (*n* = 183)	Reverter (*n* = 77)	*P* value
1) Baseline medical record assessments								
Male, n (%)	375 (61.3)	37 (46.8)	66 (45.8)	.000^**^	163 (48.5)	91 (49.7)	38 (49.4)	.963
Age, (mean ± SD)	73.15 ± 5.86_**a**_	75.24 ± 6.84_**b**_	76.53 ± 6.21_**b**_	.000^**^	76.06 ± 6.15	74.78 ± 5.55	75.95 ± 6.09	.059
Education, n (%)								
Uneducated	98 (16.0)	24 (30.4)	41 (28.5)	.000^**^	33 (9.8)	10 (5.5)	12 (15.6)	.307
0–3 years	34 (5.6)	9 (11.4)	10 (6.9)		36 (10.7)	20 (10.9)	9 (11.7)	
4–6 years	132 (21.6)	22 (27.8)	43 (29.9)		106 (31.5)	61 (33.3)	23 (29.9)	
7 - 9 years	103 (16.8)	12 (15.2)	25 (17.4)		74 (22.0)	35 (19.1)	17 (22.1)	
10 - 12 years	142 (23.2)	4 (5.1)	15 (10.4)		71 (21.1)	41 (22.4)	13 (16.9)	
13 years -	103 (16.8)	8 (10.1)	10 (6.9)		16 (4.8)	16 (8.7)	3 (3.9)	
BMI (kg/m^2^), (mean ± SD)	23.72 ± 3.20	23.59 ± 3.61	23.11 ± 3.43	.115	23.00 ± 2.96	23.24 ± 3.15	22.26 ± 3.23	.066
Risk factors of stroke, n (%)								
Hypertension	414 (67.6)	54 (68.4)	91 (63.2)	.570	212 (63.1)	116 (63.4)	55 (71.4)	.371
Diabetes Mellitus	102 (16.7)	12 (15.2)	15 (10.4)	.175	55 (16.4)	28 (15.3)	17 (22.1)	.392
Coronary heart disease	60 (9.8)	8 (10.1)	11 (7.6)	.711	27 (8.0)	16 (8.7)	5 (6.5)	.831
Atrial fibrillation	78 (12.7)	11 (13.9)	22 (15.3)	.712	59 (17.6)	27 (14.8)	10 (13.0)	.515
Hyperlipidemia	110 (18.0)	11 (13.9)	31 (21.5)	.357	39 (11.6)	19 (10.4)	12 (15.6)	.489
Obesity	79 (12.9)	12 (15.2)	11 (7.6)	.154	29 (8.6)	20 (10.9)	3 (3.9)	.185
Family history	52 (8.5)	4 (5.1)	11 (7.6)	.562	30 (8.9)	12 (6.6)	6 (7.8)	.635
Smoking, n (%)								
Current smokers	105 (17.2)	11 (13.9)	23 (16.0)	.050	67 (19.9)	27 (14.8)	19 (24.7)	.285
Former smokers	107 (17.4)	10 (12.7)	12 (8.3)		39 (11.6)	28 (15.3)	8 (10.4)	
Never smokers	400 (65.4)	58 (73.4)	109 (75.7)		230 (68.5)	128 (69.9)	50 (64.9)	
Alcohol consumption, n (%)								
None	414 (67.6)	54 (68.4)	108 (75.0)	.322	249 (74.1)	133 (72.7)	56 (72.7)	.367
Moderate	141 (23.0)	20 (25.3)	23 (16.0)		63 (18.8)	28 (15.3)	15 (19.5)	
Heavy	57 (9.3)	5 (6.3)	13 (9.0)		24 (7.1)	22 (12.0)	6 (7.8)	
2) Stroke characteristics								
Ischemic type (TOAST)								
Large-artery atherosclerosis	278 (45.4)	51 (64.6)	67 (46.5)	.052	167 (49.7)	94 (51.4)	46 (59.7)	.662
Small-artery occlusion	155 (25.3)	11 (13.9)	39 (27.1)		60 (17.9)	34 (18.6)	12 (15.6)	
Cardioembolism	89 (14.5)	5 (6.3)	17 (11.8)		59 (17.6)	28 (15.3)	7 (9.1)	
Other determined	36 (5.9)	4 (5.1)	5 (3.5)		15 (4.5)	5 (2.7)	4 (5.2)	
Undetermined ischemic stroke	54 (8.8)	8 (10.1)	16 (11.1)		35 (10.4)	22 (12.0)	8 (10.4)	
Ischemic location								
Rt. hemisphere	309 (50.5)	39 (49.4)	64 (44.4)	.394	135 (40.2)	68 (37.2)	33 (42.9)	.667
Lt. hemisphere	247 (40.4)	31 (39.2)	70 (48.6)		178 (53.0)	106 (57.9)	38 (49.4)	
Both hemisphere	56 (9.2)	9 (11.4)	10 (6.9)		23 (6.8)	9 (4.9)	6 (7.8)	
Affected level								
Cortical level	203 (33.2)	32 (40.5)	49 (34.0)	.047^*^	143 (42.6)	75 (41.0)	32 (41.6)	.729
Subcortical level	193 (31.5)	20 (25.3)	30 (20.8)		83 (24.7)	53 (29.0)	21 (27.3)	
Brainstem level	125 (20.4)	11 (13.9)	40 (27.8)		42 (12.5)	20 (10.9)	13 (16.9)	
Multiple level	91 (14.9)	16 (20.3)	25 (17.4)		68 (20.2)	35 (19.1)	11 (14.3)	
3) Treatment characteristics								
IV thrombolysis	40 (6.5)	5 (6.3)	9 (6.3)	.991	27 (8.0)	16 (8.7)	7 (9.1)	.935
IA thrombolysis	9 (1.5)	2 (2.5)	3 (2.1)	.722	20 (6.0)	4 (2.2)	1 (1.3)	.049^*^
IV heparin	52 (8.5)	5 (6.3)	9 (6.3)	.575	29 (8.6)	10 (5.5)	8 (10.4)	.301
Antiplatelet agent	485 (79.2)	65 (82.3)	115 (79.9)	.818	239 (71.1)	131 (71.6)	53 (68.8)	.901
Rehabilitation Therapy	157 (25.7)	25 (31.6)	43 (29.9)	.363	133 (39.6)	54 (29.5)	36 (46.8)	.015^*^
Cognitive Therapy	26 (4.2)	4 (5.1)	5 (3.5)	.844	19 (5.7)	11 (6.0)	10 (13.0)	.061
4) Neuropsychological assessments								
Cognitive function (K-MMSE)								
3 months from onset	26.86 ± 3.41_**a**_	23.18 ± 3.69_**b**_	25.85 ± 3.82_**c**_	.000^**^	14.46 ± 9.71	15.95 ± 7.64	16.08 ± 7.09	.112
12 months from onset	26.88 ± 3.50_**a**_	27.01 ± 3.30_**a**_	20.66 ± 4.90_**b**_	.000^**^	14.68 ± 9.95_**a**_	22.46 ± 5.78_**b**_	10.52 ± 7.21_**c**_	.000^**^
Variation (from 3 months to 12 months)	0.03 ± 1.17_**a**_	3.84 ± 1.31_**b**_	−5.19 ± 3.05_**c**_	.000^**^	0.21 ± 1.10_**a**_	6.51 ± 4.03_**b**_	−5.56 ± 2.99_**c**_	.000^**^
Stroke Severity, ADL, Motor, Gait, Depression, QoL (3 months from onset)								
NIHSS	0.78 ± 1.98_**a**_	1.15 ± 2.06_**a**_	1.32 ± 2.15_**b**_	.009^**^	5.75 ± 7.43_**a**_	3.17 ± 4.69_**b**_	5.08 ± 4.68_**b**_	.000^**^
mRS	1.12 ± 1.12_**a**_	1.51 ± 1.32_**b**_	1.63 ± 1.25_**b**_	.000^**^	2.79 ± 1.68_**a**_	2.37 ± 1.54_**b**_	3.26 ± 1.29_**a**_	.000^**^
FIM	117.40 ± 15.58_**a**_	113.08 ± 18.30_**ab**_	111.16 ± 19.83_**b**_	.000^**^	83.68 ± 39.78_**a**_	95.74 ± 32.33_**b**_	81.34 ± 31.60_**a**_	.001^**^
K-MBI	93.31 ± 16.11_**a**_	89.09 ± 17.82_**ab**_	87.34 ± 19.10_**b**_	.000^**^	64.13 ± 38.83_**a**_	75.71 ± 30.51_**b**_	61.61 ± 32.50_**a**_	.001^**^
FMA (affected side)	93.92 ± 16.11	90.53 ± 19.31	91.44 ± 17.83	.094	70.49 ± 36.80_**a**_	81.45 ± 30.54_**b**_	65.90 ± 35.97_**a**_	.000^**^
FAC	4.51 ± 1.05_**a**_	4.15 ± 1.40_**b**_	4.00 ± 1.35_**b**_	.000^**^	2.83 ± 2.10_**a**_	3.45 ± 1.79_**b**_	2.70 ± 1.87_**a**_	.001^**^
GDS	5.40 ± 3.79_**a**_	6.83 ± 4.19_**b**_	7.41 ± 4.35_**b**_	.000^**^	7.63 ± 4.09_**a**_	8.03 ± 4.27_**ab**_	9.65 ± 4.04_**b**_	.022^*^
EQ-5D	0.76 ± 0.29_**a**_	0.67 ± 0.34_**b**_	0.63 ± 0.36_**b**_	.000^**^	0.39 ± 0.41_**a**_	0.49 ± 0.40_**b**_	0.31 ± 0.37_**a**_	.003^**^

*n*, Number; *SD*, Standard Deviation; *BMI*, Body Mass Index; *TOAST*, Trial of Org 10172 in Acute Stroke Treatment; *Rt*, Right; *Lt*, Left; *IV*, Intra-Venous; *IA*, Intra-Artrial; *K*-*MMSE*, Korean Mini Mental State Examination; *NIHSS*, National Institutes of Health Stroke Scale; *mRS*, modified Rankin Scale; *ADL*, Activity of Daily Living; *FIM*, Functional Independence Measure; *K-MBI*, Korean version of Modified Barthel Index; *FMA*, Fugl-Meyer Assessment; *FAC*, Functional Ambulation Categories; GDS, Geriatric Depression Scale; QoL, Quality of Life; EQ-5D, EuroQol-5D

^*^ *p* < 0.05; ^**^ *p* < 0.01

_**abc**_ Post HOC group

## Discussion

In our study, total the percentage of cognitive impairment group did not change at 12 months compared to 3 months assessment. Otherwise, the percentage of cognitive impairment at 3 months, the percentage of patients in the reverter group, and the percentage of patients transferring from the NCG to the ICG at 12 months were higher in older patients compared to total group analysis (Table 2) (Fig. 2). Influencing factors for delayed cognitive change were discretely determined in the NCG and ICG of total and older patients. Hypertension history and onset age, sex, education level were somewhat repeated influencing factors. Although presence of atrial fibrillation, smoking, alcohol history showed statistical significance, large difference of number of patients between groups made it difficult to define it as meaningful result. Another unique aspect of our study is that we included functional assessment since it could influence on patients’ delayed cognitive function. Patients with better functional assessment scores not only in cognitive field but in all other domains including activities of daily living, motor function, mobility and gait, general functional level, quality of life at 3 month tend to have less cognitive decline and more cognitive improvement from 3 month to 12 month. Otherwise, stroke characteristics including ischemic type, location, treatment characteristics showed no significant difference between the groups. Patients in the ICG-RG that were aged >65 years received more rehabilitation therapy. Moreover, compared to the other patient groups, the patients in the ICG-SG received less cognitive therapy, which was a component of the rehabilitation therapy. We believe that unlike the other factors, the administration of rehabilitation treatment depended on the cognitive status of the patient; however, rehabilitation was not a factor that improved cognitive function.

Stroke severity, onset age, pre-stroke cognitive function, level of education, and bilateral lesions are well-known factors associated with development of post-stroke dementia [33–36]. In contrast, a cohort study of younger stoke patients (mean age, 60 years) showed that more than 30% of the patients with mild cognitive impairments between 0 and 6 months were classified as cognitively intact by 12 to 18 months [16]. For older patients (mean age, 80.4 ± 3.8), about 50% of the patients experienced an improvement in MMSE at 15 months [17]. As the prognosis of stroke varies according to the patient’s age at onset, identifying the factors affecting cognitive changes by age might aid in both preventing secondary cognitive decline, and enhancing post-stroke cognitive function. In our study, separating patients by age, cognitive function at 3 month, and aspect of cognitive change to find the propensity of each prevalence and influencing factors was meaningful. Also, compared to other cross sectional studies, our study analyzed delayed post stroke cognitive function and focused on amount and aspect of cognitive change from 3 month to 12 month and its influencing factors.

To carry this analysis further, we examined the differences in the MMSE scores among the groups by using the cutoff score of <24 points, conventionally accepted for the diagnosis of significant cognitive impairment, i.e., dementia [37]. Previous studies have established more than 3 points of MMSE variability as a significant change for improvement or decline in cognitive function.

MMSE is the most frequently applied test for dementia screening. A systematic review and meta-analysis examining cognitive tests to detect dementia found 10,263 cases of dementia identified from 36,080 participants in 108 cohort studies. The result reported a sensitivity of 0.81 and a specificity of 0.89 for the MMSE [38]. The MMSE requires only 5–10 min to evaluate various cognitive domains (orientation, memory, language, attention, visuospatial) and is practical to use serially and routinely [39].

The overall prevalence of dementia in subjects aged greater than 65 in Korea is estimated to be 9.2%. In addition, the pooled age-specific prevalence of dementia is estimated to increase with each 5-year age band (65–69 years) [40]. This result is much higher than the estimated overall prevalence of dementia in Asian people [41]. We analyzed our data by separating patients older than 65 y/o to compare the age factors. The percentages of normal cognitive group and cognitive impaired patients and mean MMSE scores showed significant differences between total and older patient groups. In particular, the percentage of patients in the reverter group was higher and the converter group was lower at 12 months in older patient group, and their average MMSE showed differences by age. Otherwise, in older ICG, less factors were significant compared to other groups. This finding may be due to the lower percentage of patients when compared to the entire study population and NCG.

## Limitations

First, we only used a MMSE to test cognitive function. Although there are 40 other more detailed tests for dementia diagnosis in healthcare settings, we required multi-domain, serial functional assessments for screening and detecting post stroke cognitive decline. Also, MMSE was optimal for our insurance benefits and medical policy which can be done consecutively for our large-scale cohort study [40].

Second, we excluded patients with pre-stroke cognitive decline, but we had no objective assessment data on which to base our exclusions. Instead, patients’ pre-stroke cognitive function was determined by administered questionnaires and face-to-face interviews. Additional studies, such as volumetric analysis by MRI/MRA scans, may be valuable to investigate and compare the severity of stroke among the groups.

Third, we excluded patients who are not capable of 1 year follow up examination including functional assessments which could make selection bias. However, it could be a strength of this cohort study which differs from others and it may suggest more objective data for stroke survivors.

## Conclusions

The prevalence of cognitive impairment at 3 month showed difference between total and older patient groups. To analyze the cognitive change from 3 month to 12 month, the proportion stable group was dominant in NCG and converter group was higher in ICG. By investigating the influencing factors from each group, we were able to identify the early predictors including the age factor.

## Abbreviations

CG: Converter group
EQ-5D: Euro quality of Life
FAC: Functional ambulatory category
FIM: Functional independence measure
FMA: Fugl-meyer assessment
GDS-SF: Geriatric depression scale-short form
ICG: Impaired cognitive group
K-MBI: Korean modified Barthel index
K-MMSE: Korean mini-mental State examination
K-NIHSS: Korean national institute of health stroke scale
KOSCO: Korean stroke cohort for functioning and rehabilitation
mRS: Modified rankin scale
NCG: Normal cognitive group
RG: Reverter group
SG: Stable group
